# Enhancement of conservation knowledge through increased access to botanical information

**DOI:** 10.1111/cobi.13291

**Published:** 2019-02-26

**Authors:** Cátia Canteiro, Laísa Barcelos, Fabiana Filardi, Rafaela Forzza, Laura Green, João Lanna, Paula Leitman, William Milliken, Marli Pires Morim, Kristina Patmore, Sarah Phillips, Barnaby Walker, Marie‐Hélène Weech, Eimear Nic Lughadha

**Affiliations:** ^1^ Royal Botanic Gardens, Kew TW9 3AE Richmond Surrey U.K.; ^2^ Universidade Federal de Pelotas Faculdade de Agronomia Eliseu Maciel Avenida Eliseu Maciel, s/n ‐ Capão Leão 96050–500 Capão do Leão RS Brazil; ^3^ Jardim Botânico do Rio de Janeiro Pacheco Leão 915 22460−030 Rio de Janeiro RJ Brazil

**Keywords:** Brazil, conservation impacts, digitization, extinction risk assessments, flora, knowing‐doing gap, natural history collections, Reflora, virtual herbarium, Brasil, colecciones de historia natural, digitalización, flora, herbario digital, impactos de la conservación, Reflora, vacío entre saber y hacer, valoraciones del riesgo de extinción

## Abstract

Herbarium specimens are increasingly recognized as an important resource for conservation science and virtual herbaria are making specimens freely available to a wider range of users than ever before. Few virtual herbaria are designed with conservation use as a primary driver. Exceptionally, Brazil's Reflora Virtual Herbarium (RVH) was created to increase knowledge and conservation of the Brazilian flora. The RVH is closely integrated with the Flora of Brazil 2020 platform on which Brazil's new national Flora is under construction. Both resources are accessible via the Reflora home page and thousands of users move seamlessly between these Reflora resources. To understand how the Reflora resources are currently used and their impact on conservation science, we conducted a literature review and an online survey. We searched for publications of studies in which Reflora resources were used and publications resulting from Brazilian researchers who were part of Reflora's research and mobility program. The survey contained multiple choice questions and questions that required a written response. We targeted Reflora webpage visitors with the survey to capture a wider range of Reflora users than the literature review. Reflora resources were used for a variety of conservation‐relevant purposes. Half the 806 scientific publications in which Reflora was cited and 81% of the 1069 survey respondents accessing Reflora resources mentioned conservation‐relevant research outputs. Most conservation‐relevant uses of the Reflora resources in scientific publications were research rather than implementation focused. The survey of Reflora users showed conservation uses and impacts of virtual herbaria were more numerous and diverse than the uses captured in the literature review. Virtual herbaria are vital resources for conservation science, but they must document use and impacts more comprehensively to ensure sustainability.

## Abstract 2

*Article impact statement*: Virtual herbaria, vital resources for taxonomy, conservation and ecology, must showcase their relevance to gain ongoing support.

## Abstract (es)

Revalorización del Conocimiento sobre Conservación por Medio de Mayor Acceso a la Información Botánica

**Resumen**

Los especímenes de los herbarios son reconocidos cada vez más como un recurso importante para la ciencia de la conservación, y, como nunca antes, los herbarios virtuales están poniendo a los especímenes a libre disposición para una gama mucho más amplia de usuarios. Pocos herbarios virtuales están diseñados con el uso para la conservación como conductor primario. Excepcionalmente, se creó el Herbario Virtual Reflora de Brasil (RVH, en inglés) para incrementar el conocimiento y la conservación de la flora brasileña. El RVH está integrado cuidadosamente con la plataforma Flora de Brasil 2020, sobre la cual está bajo construcción la nueva flora nacional de Brasil. Se accede a ambos recursos a través de la página inicial de Reflora y miles de usuarios navegan continuamente entre estos recursos de la página. Para entender cómo se usan actualmente los recursos de Reflora así como su impacto sobre la ciencia de la conservación, realizamos una revisión de la literatura y una encuesta en línea. Buscamos las publicaciones de estudios que usaron los recursos de Reflora y las publicaciones elaboradas por investigadores brasileños que formaron parte del programa de investigación y movilidad de Reflora. La encuesta incluyó preguntas de opción múltiple y preguntas que requerían una respuesta escrita. Enfocamos la encuesta en los visitantes a la página web de Reflora para obtener una gama más amplia de usuarios de Reflora que la que obtendríamos sólo con la revisión de la literatura. Los recursos de Reflora se usaron para una variedad de propósitos relevantes para la conservación. La mitad de las 806 publicaciones científicas en las que se citó a Reflora y el 81% de los 1069 encuestados que acceden a los recursos de Reflora mencionaron resultados de investigación relevantes para la conservación. La mayoría de los usos relevantes para la conservación de los recursos de Reflora en las publicaciones científicas estuvieron más enfocados en la investigación que en la implementación. La encuesta para los usuarios de Reflora mostró que los usos e impactos de los herbarios virtuales fueron más numerosos y diversos que los usos capturados en la revisión de la literatura. Los herbarios virtuales son recursos vitales para la ciencia de la conservación, pero deben documentar su uso y sus impactos exhaustivamente para asegurar su sustentabilidad.

## Introduction

Herbarium specimens are increasingly highlighted as an important resource in conservation science. Each specimen is evidence of the presence of a species at a particular location and time. When accurately identified and interpreted, specimens provide baseline information about the distribution of individual plant species and often the assemblage of species occurring at a location. Although most herbaria were established, developed, and curated with taxonomy and systematics as primary motivations, their potential for use in conservation has been recognized for decades, and recently the range of documented conservation uses for specimens has grown rapidly (Lavoie 2013; Greve et al. 2016; Nualart et al. 2017).

The central role of herbarium data in evaluating extinction risk in plants is well‐defined (Willis et al. 2003), documented (e.g., Rivers et al. 2010), and tested (Rivers et al. 2011). For most tropical plants, herbarium specimens are the primary basis for conservation status assessments. They enable application of the International Union for Conservation of Nature's (IUCN) criterion B (IUCN 2012); observations of the duration and detail required to apply other criteria are rarely available except for the most widespread and well‐known tropical species (Brummitt et al. 2015). Another long‐standing practical conservation application is the use of phenological data from herbarium specimens to schedule fieldwork coinciding with fruiting periods of species targeted for seed collection for ex situ conservation (Lindsay 2007). Such activities are often undertaken by scientists based at botanical gardens or museums that house major herbaria and stimulated by the Global Strategy for Plant Conservation (GSPC) of the United Nations’ Convention on Biological Diversity (CBD 2018). However, large‐scale digitization, dissemination, and aggregation of herbarium data over the past 2 decades (MNHN 2018; Naturalis Biodiversity Center 2018) have effectively opened herbaria to the wider global scientific community, which previously had limited and often expensive access, and to myriad other stakeholders who previously had little or no access.

The primary aim of most of the herbarium digitization initiatives is increasing access to herbarium specimens (Nic Lughadha & Miller 2009). However, drivers for specimen digitization differ widely between initiatives, and motivations may evolve substantially over the extended delivery periods of major digitization projects. Consequently, eventual uses of the resulting virtual herbaria (VH) may differ from the applications envisaged by those conceiving and designing them. For example, original motivations for creation of Australia's Virtual Herbarium (AVH) were opportunities to improve collection management and streamline taxonomic processes, but these uses are now greatly exceeded by use for ecological research (Cantrill 2018).

Unlike earlier herbarium digitization initiatives, the Reflora Virtual Herbarium (RVH) (http://reflora.jbrj.gov.br/reflora/herbarioVirtual/) had conservation as a primary objective from its inception. Brazil's Reflora program was established by the Brazilian Government in 2010 “to retrieve and make available images and information concerning Brazilian plants deposited chiefly in overseas herbaria” and “to increase knowledge and conservation of the Brazilian flora” (CNPq 2010; Nic Lughadha et al. 2016). The RVH is hosted and managed by Rio de Janeiro Botanical Garden (JBRJ). Consistent with its twin foci of knowledge and conservation, the Reflora program grew to encompass redevelopment and maintenance of the online List of Brazilian Flora, hosted by JBRJ and originally funded by the Brazilian National Center for Plant Conservation (CNCFlora). The remit of CNCFlora is to generate, coordinate, and disseminate information concerning the biodiversity and conservation of Brazil's threatened flora. The RVH and List of Brazilian Flora became intimately linked because many of the specimen images in RVH serve as vouchers evidencing species distribution information in the List of Brazilian Flora. Recently, this list has been redeveloped and rebranded as Flora of Brazil 2020, the platform on which Brazil's online Flora is under construction by over 800 botanical specialists. Their goal is a complete treatment of the Brazilian flora by 2020, meeting GSPC target 1 at the national level and providing the foundation to deliver target 2, a conservation assessment for all known plant species (CBD 2018). The CNCFlora is the leading Brazilian entity undertaking extinction‐risk assessments of endemic species.

In Reflora's first phase, supported by Brazilian organizations, intensive specimen digitization activities focused on Brazilian specimens deposited at the Royal Botanic Gardens, Kew (K), and the National Museum of Natural History, Paris (P), were complemented by researcher mobility funding, which enabled >86 Brazilian researchers to access the European collections in person. In the second phase, initiated in 2014, the Reflora program received support related to Brazil's new Global Biodiversity Information Facility node (SiBBr) that enabled participation of other European and US herbaria and mobility funding for postgraduates. Also in 2014, Brazilian herbaria began dissemination of their images and data via RVH (supported by Brazil's National Forest Inventory).

Recognizing the value of Reflora, the UK government's Newton Fund supported continuation of UK digitization efforts and researcher visits into early 2016. By late 2016, Reflora was widely recognized as successful. Its online resources provide vital information that supports national environmental policies and management of natural resources and enables the United Kingdom and Brazil to meet their CBD obligations and make progress toward GSPC targets. Stakeholders emphasize that many of the benefits, including research publications, are still to come (Grimes & McNulty 2016).

As a result of these concerted efforts, resources now identified as products (wholly or partially, directly or indirectly) of the Reflora program include RVH, with >3 million images of plant specimens; Flora of Brazil 2020, with 46,648 species listed; and scientific publications of many Brazilian researchers who benefited from the Reflora program's research and mobility funding.

Images in RVH are from 53 herbaria in Brazil, Europe, and the United States and are primarily of specimens collected in Brazil, but also include specimens from other Neotropical countries, especially nomenclatural types and historical collections. Key specimen data have been transcribed to enable searches by scientific name, collector, collector number, collection locality, and date. The RVH contains specimens of approximately 93% of flowering plant species listed for Brazil and 100% of genera and families. The 3.1 million specimens represented in RVH encompass material from all Brazilian states and equate to 0.36 specimens/km^2^, about 1 in 3 of the 1.08 specimens/km^2^ estimated for the Brazilian flora (Morim & Nic Lughadha 2015).

**Figure 1:**
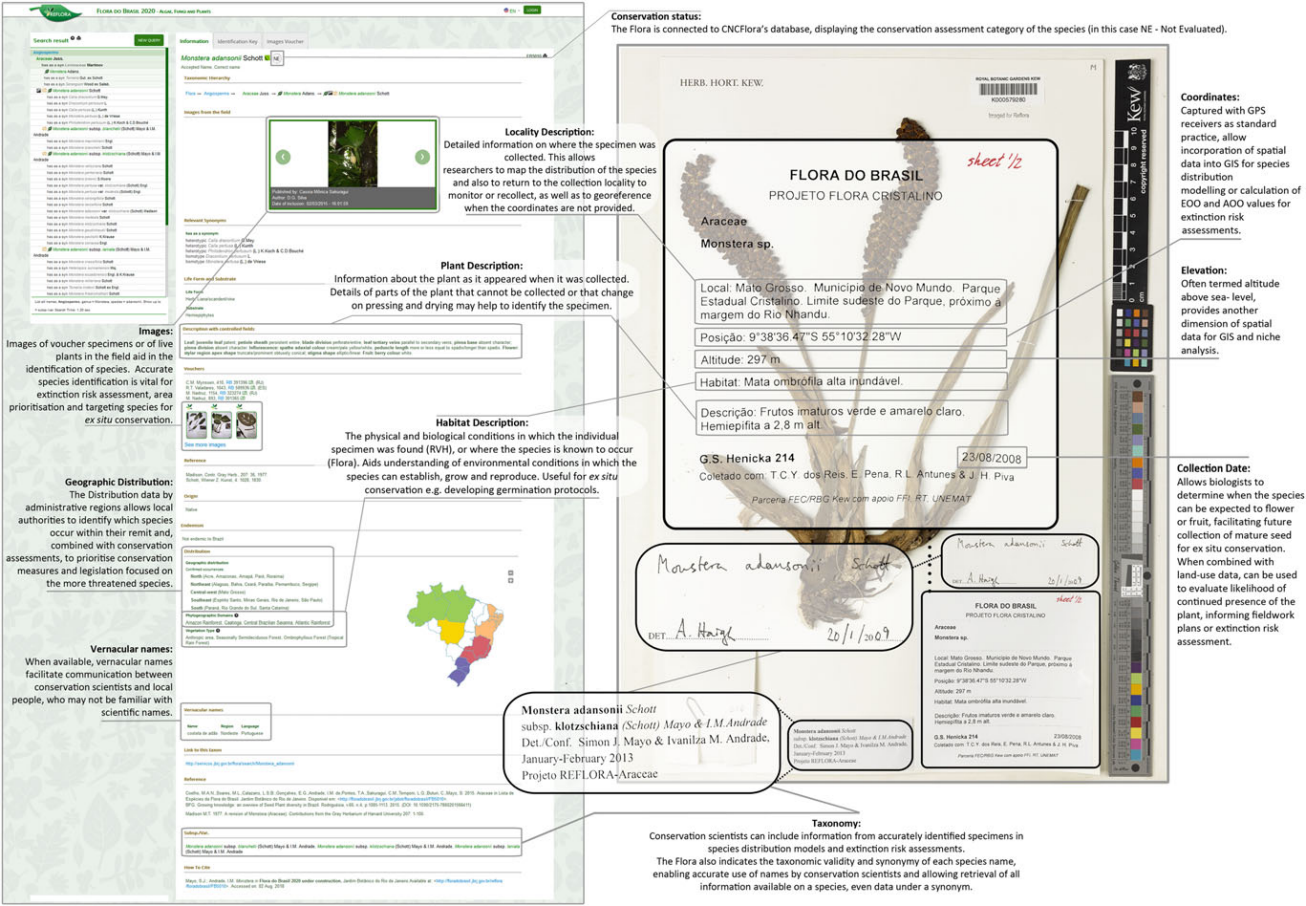
Example of a virtual herbarium specimen from Reflora Virtual Herbarium and species profile from the Flora of Brazil highlighting data valuable for conservation science and some conservation applications (EOO, extent of occurrence; AOO, area of occupancy).

Flora of Brazil 2020 includes accepted names and synonyms for all plant and fungal species known to occur in Brazil and standardized information on their status (native or non‐native), distribution within Brazil (by region, state, biome, and habitat), and links to voucher specimens, in RVH or elsewhere, that underpin the reported distribution. For a growing proportion of the accepted species (34%, BFG 2018), Flora of Brazil 2020 includes detailed morphological information combining standardized and free‐text descriptors (e.g., nonstandard observations on morphology or taxonomy) sufficient to distinguish each species from all others documented. For smaller proportions of plant species, Flora of Brazil 2020 also includes links to extinction‐risk assessments (16%) (Martins et al. 2018), of which 91% are supported by specimens in the RVH. Reflora resources offer potential for a wide range of other conservation applications such as national action plans and recognition of key biodiversity areas (Giulietti et al. 2009) (Fig. 1).

We sought to determine quantitatively and qualitatively, how Reflora resources, especially herbarium data, are used to attain the original objectives of the Reflora program: increase knowledge and conservation of Brazilian flora. Information on the use of RVH illustrates the role of herbarium specimens in conservation research. The use of RVH cannot be considered in isolation from Flora of Brazil 2020 because Flora of Brazil contents rely heavily on the specimen data in RVH; many researchers use both resources simultaneously.

## Methods

### Literature Review

In September 2017, we searched Google Scholar with Publish or Perish (PoP) software (Harzing 2007). We searched for the term *reflora*. Our objective was to include all papers that used RVH and publications by Brazilian researchers funded through Reflora's research and mobility program, most of whom consulted, annotated, and supported digitization of herbarium collections held at Kew and Paris or both. Because of variations in citation practices over time, authors of some of the papers returned by our search probably used only Flora of Brazil 2020. Thus, our initial data set was as comprehensive as possible with respect to RVH but included only a fraction of the literature in which Flora of Brazil was cited (not the main focus of our study). We excluded papers published before 2010 because they predated establishment of Reflora.

A Portuguese speaker evaluated papers in Portuguese. We determined which sections of each paper contained *reflora* (e.g., methods, acknowledgements, or references). Papers citing Reflora in methods were selected for further analysis. We searched these papers for *conserv* in the text of the paper and determined whether the paper was relevant to conservation. We excluded papers in which *conserv* appeared in a context other than biodiversity conservation (e.g., papers on niche conservatism), but papers in which authors articulated the actual or potential relevance of their work to biodiversity conservation were tagged as conservation relevant, as were papers reporting plant diversity of protected areas because knowledge of plants in Brazil's protected areas is lacking, which impedes their conservation (JBRJ 2018). We ascertained the foci of each paper from the title and abstract. We also tagged for further analysis papers reporting new species, new genera, rediscoveries, or first records because such studies have high conservation relevance in a megadiverse country where the plant inventory is far from complete (Sousa‐Baena et al. 2014) and earlier analyses show elevated levels of extinction risk among newly discovered and rediscovered species (Pimm et al. 2010; Martinelli et al. 2018). Thus, conservation relevance of a paper was determined such that included papers made more than a passing reference to conservation. We assigned a publication type (e.g., thesis, poster, and article).

To ensure consistency between paper reviewers, we used Fleiss’ kappa test. All 4 reviewers independently tagged the same random set of 25 papers. We calculated the kappa value to quantify agreement between reviewers. To improve the initial score of 0.65, we compared and standardized application of tags (Supporting Information) and determination of conservation relevance. On a second random set of papers the score was 0.77, indicating substantial levels of agreement (Landis & Koch 1977). Reviewers analyzed subsets of papers consistent with their language skills and scientific experience.

We analyzed papers mentioning Reflora in their Methods to determine, if possible, which Reflora resource was consulted (RVH, Flora of Brazil 2020, List of Brazilian Flora) and what information was gathered and how it was used. We analyzed papers identified as conservation relevant to determine their focus and assigned them to research fields.

We listed published new species, new genera, first records, and rediscoveries from papers that cited Reflora and the country where species were found. For Brazilian species, we also recorded state and locality. We recorded conservation status, if stated in the paper. For new taxa, we recorded year of first collection and type specimen collection and locality. For rediscoveries, we noted year of rediscovery, previous last record, and type specimen collection and locality. We evaluated proportions of new species, first records, and rediscoveries with conservation assessments and compared proportions of species in different conservation categories.

### Online Survey

We posted an online survey for 1 month on the Reflora website at points visible to casual and logged‐in users of RVH and the Reflora homepage. The survey was in Portuguese and English (Supporting Information). Our intention was to target all users of RVH while minimizing inclusion of users consulting only Flora of Brazil 2020, although complete separation was not feasible due to close integration of the 2 systems. The main aim of the survey was to ascertain if and how visitors to RVH used the site for conservation‐relevant purposes. Response rates exceeded expectations, so we confined our analyses to responses received from 10 to 20 October 2017. To check that this 11‐day subsample was representative of responses collected over the month, we compared answers to the multiple choice questions across the entire period. Survey responses were read and tagged using the same schema as the literature review (Supporting Information). Where conservation relevance was declared in 1 of the multiple choice responses, we sought supporting evidence in the free‐text response to apply the appropriate tag. We compared numbers of survey responses with Google Analytics numbers for unique users of RVH and Flora of Brazil 2020.

## Results

### Literature Review

Our PoP search returned 955 items after deduplication. We excluded 149 items from further analysis because they were either published before 2010 (55); unrelated to Brazil's Reflora program (58); in a language we could not read (1); not a publication (e.g., citation or blog [32]); or not accessible (3).

Of the remaining 806 papers, most mentioned Reflora in references (444), followed by acknowledgments (230) and methods (109). Papers mentioning Reflora in methods used the resources mainly to check nomenclature (51), identify plant species (35), or confirm their distribution (24). Approximately 33% stated herbarium specimens in RVH had been consulted, and 6% implied such use.

**Figure 2:**
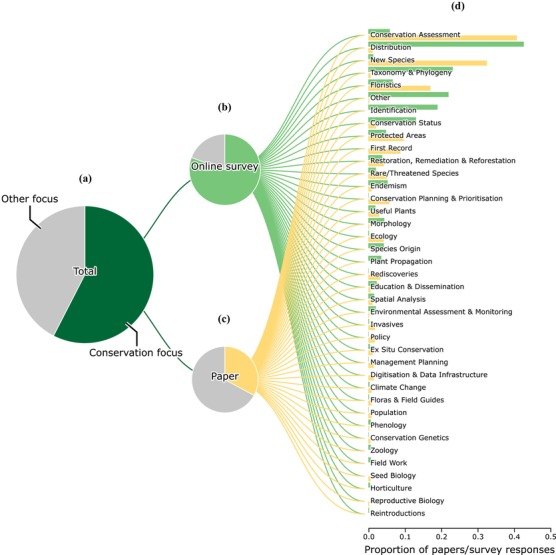
Overview of uses of Reflora resources based on a literature review and online survey: proportion of (a) all items, (b) online survey responses, (c) scientific papers with conservation‐relevant uses, and (d) papers and survey responses associated with a particular use.

We identified 337 publications that could be relevant for conservation. We excluded theses and posters as unlikely to be consulted by policy makers or conservation practitioners, leaving 269 for in‐depth analysis. Most of these papers assessed the conservation status of species (134), described new species (107), presented floristic studies or vegetation surveys (56), often in protected areas (32), or included more than 1 of these items (Fig. 2). Thirty papers had a primary focus on conservation.

**Figure 3:**
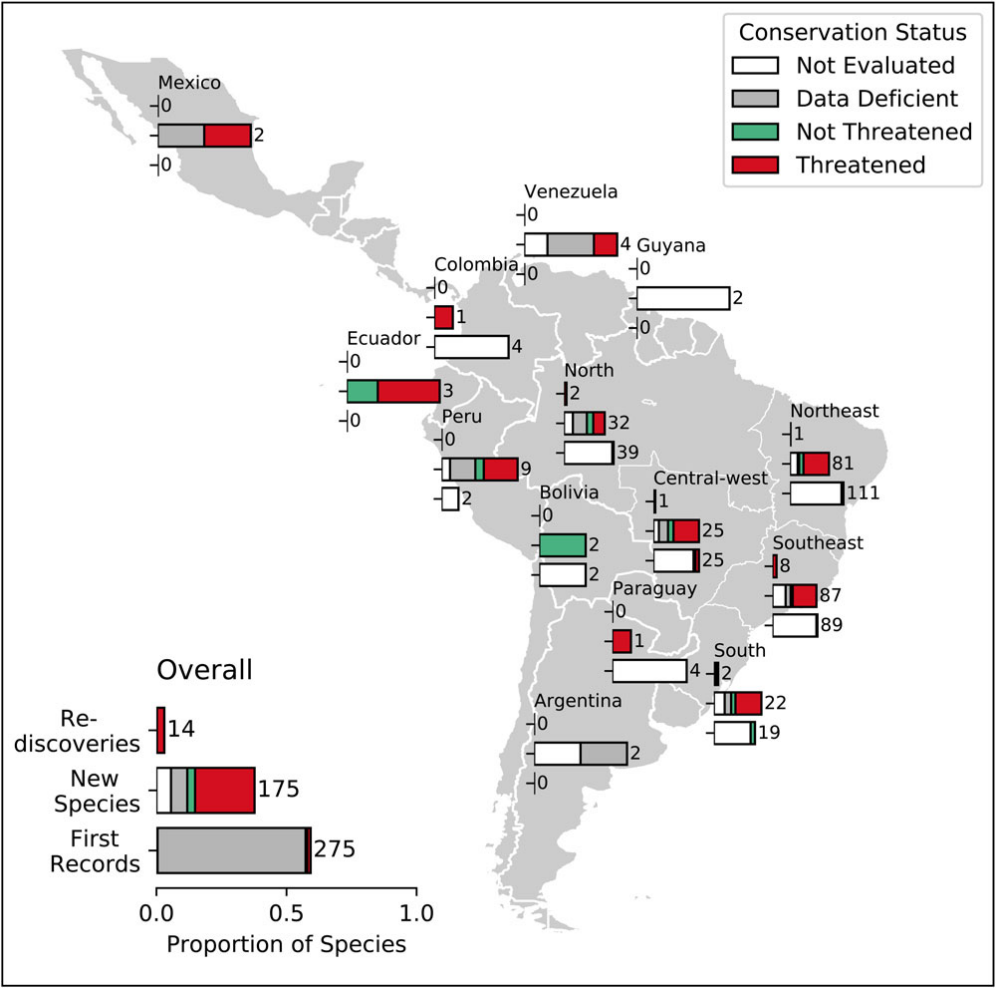
Distribution of species documented as rediscovered, described as new to science, or first reported for a particular area and the conservation status of these species by region as reported in publications based on research that included use of Reflora.

Most species new to science, rediscoveries, and first records were Brazilian, but the data set also included 29 species (new taxa and first records) representing additions to the flora of 8 other South American countries and Mexico (Fig. 3). Most papers reporting extra‐Brazilian records involved authors who participated in Reflora's researcher mobility program, which enabled study of Brazilian collections in a broader geographical context. Brazil had 281 first records for particular areas, of which 36 were national first records, including 1 African species (Alves & Roque 2016). A single paper (Maia et al. 2015), reporting 2111 fungi added to the mycota known from Brazil was excluded from analysis because distribution data were incomplete.

Taxa new to science from Northeast or Southeast Brazil, collectively accounted for 72% of those in the whole data set, and the majority were published with conservation assessments deeming them threatened. First records of taxa for a state or region were also predominantly in Northeast and Southeast Brazil, but most lacked conservation assessments. Additions to the flora of South and Central‐west Brazil were fewer but had similar proportions evaluated for conservation status and deemed threatened. Species assessed as data deficient accounted for a greater proportion of new species described for North Brazil than for other regions. Most rediscovered species were from Southeast Brazil, all threatened (Fig. 3). Of 14 rediscoveries reported, 6 species were rediscovered over 100 years since last recorded.

**Figure 4:**
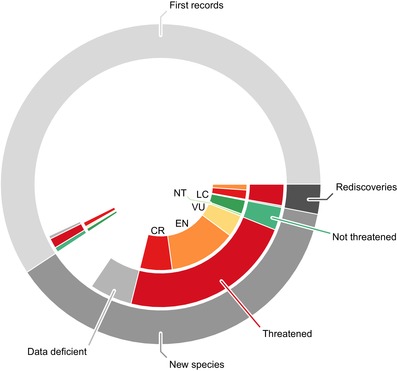
Relative number (464) of species reported in the literature review of publications based on research that included use of Reflora as new species, rediscovered species, and first reports and the proportion of each assessed as threatened, not threatened, data deficient, least concern (LC), near threatened (NT), vulnerable (VU), endangered (EN), and critically endangered (CR).

The extent to which new species, rediscoveries, and first records were published with a conservation assessment and their conservation category spectra differed between regions (Fig. 4). All but 1 report of a rediscovered species included a conservation assessment indicating that the species were threatened. Most were categorized as critically endangered; the remainder were categorized as endangered. New species were usually accompanied by a conservation assessment (84% assessed), and most of those assessed were threatened (73%). The number of species evaluated as data deficient (18%) exceeded the number of species evaluated as not threatened (10%). Among new species evaluated as threatened, most were endangered (56%). New species evaluated as critically endangered (26%) outnumbered those deemed vulnerable (18%).

### Online Survey

We analyzed all 1069 survey responses received in the first 11 days. Selecting, from predefined options, areas of their work for which they consulted Reflora resources, most respondents selected taxonomy (78%), ecology (42%), or conservation (41%). Totals exceeded 100% because many users selected more than 1 area (Supporting Information). However, in responding to a subsequent, conservation‐focused question, 81% of respondents reported using Reflora resources for their conservation‐related work. Analysis of their more detailed, free‐text answers showed that most sought distribution (44%), taxonomy (22%), identifications (20%), or the conservation status of species (14%). Conservation‐relevant uses, determined by analysis of free‐text responses, were particularly evident among lower‐frequency responses such as floristics (7%), conservation assessments (6%), protected areas (6%), rare or threatened species (6%), and endemism (6%). Overall levels of RVH use among respondents appeared broadly comparable to those from the literature. Approximately, 27% stated they had consulted the herbarium specimens available in RVH and 14% implied it.

**Figure 5:**
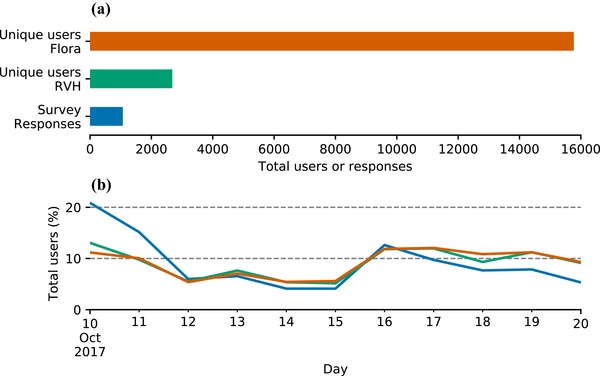
Over 11 days, the (a) number of unique users of Flora of Brazil and Reflora Virtual Herbarium (RVH) and (b) the percentage of the total number of users.

Google Analytics data showed that unique users of the Flora of Brazil 2020 (15,771) exceeded those of RVH (2681) about 6‐fold over the period analyzed, but daily changes were similar, consistent with users moving seamlessly between systems (Fig. 5). Survey response rates were highest on the first day, tracked numbers of unique users for 1 week, and then declined.

## Discussion

Our inclusive concept of conservation‐relevance encompassed papers mentioning potential applications of their results to conservation, even if not the primary focus, and responses to an online survey that indicated platform users’ work was conservation relevant even if links to conservation were not clearly articulated. Each of these sources has limitations and potential biases, but they are complementary, so collectively they provide an early snapshot of conservation use. Although the conservation focus is sharper in some areas than others, our results are of interest because ours is the only study of its kind.

### Conservation Relevance, Use, and Impact

Many Reflora users are conscious of the actual or potential conservation relevance of their actions and publications. About half of the scientific publications referencing Reflora were identifiable as conservation relevant, and over 80% of the survey respondents declared their work was conservation relevant. These proportions may be inflated due to self‐selection by our respondents (discussed below) and our inclusive definition of conservation relevance. Nonetheless, they represent encouraging evidence that a resource established with conservation as a primary objective is accessed and consulted by many conservation‐aware users. However, conservation awareness and growth in conservation‐relevant knowledge are not equivalent to conservation impact (Knight et al. 2008). The challenge represented by the research‐implementation gap (or the science‐practice gap) is much debated in conservation science (Bertuol‐Garcia et al. 2018).

The Reflora program increased conservation‐relevant information available for the flora of Brazil and other Latin American countries. Two high‐profile botanical publications of 2017 would not have been possible without Reflora resources. For the first time, the plant diversity of Amazonia's lowland rain forest is quantified based on a taxonomically verified species list underpinned by voucher specimens identified by specialists (Cardoso et al. 2017). A second landmark publication lists all known native New World vascular plant species, the first catalogue of the plant diversity of the Americas (Ulloa Ulloa et al. 2017). Each paper clearly articulates its conservation significance in terms of GSPC delivery or the need for more collections and geographic and abundance data to understand species distribution patterns and find undescribed species. Both papers are cited in publications highlighting irreplaceable biodiversity of areas threatened by mining (Moraes 2018; Pérez‐Escobar et al. 2018; Roy et al. 2018) and will provide fundamental resources for conservation assessment and planning for years to come.

These synthesis papers are clearly conservation relevant, but they lie on the science side of the science‐practice gap, perhaps inevitably when documenting tens of thousands of plant species. At the other extreme, focusing on individual species, the rediscovery of a Neotropical rheophyte (Bove & Philbrick 2014) represents a stand‐out example of scientists striving to close the research‐implementation gap so as to maximize prospects for conservation action. *Podostemum flagelliforme* was known only from the type specimen, collected in 1844, until its 2013 rediscovery in Tocantins. Before publishing the authors contacted the relevant Brazilian governmental agency (ICMBio) to argue for boundary adjustments to a proposed ecological corridor so as to include the only known population of this unique plant. Few similar actions were documented in papers in our literature review, but collectively the conservation assessments included in taxonomic treatments made an important contribution to knowledge of Brazil's most threatened species. If the conservation impact of Reflora resources were to be judged purely from scientific publications that cite them, one would conclude that Reflora impacts are predominantly on the knowing rather than the doing side of the science‐practice gap.

However, results from other facets of our study reveal different perspectives on Reflora uses. Comparison of use cases reported in survey responses with those in scientific publications suggests that certain types of Reflora use are less likely to be documented as such in publications. For example, the most frequent Reflora use described by respondents was the study of distribution (users checked distribution or frequency of particular species) with summary data in Flora of Brazil 2020 and location data in RVH. In contrast, relatively few scientific publications had distribution or frequency per se as a main focus, but a large proportion had conservation assessment as a main focus (Fig. 2), and most such assessments undertaken in Brazil are based primarily on distribution data from herbarium specimens (Souza et al. 2016).

### Data Comprehensiveness and Representativeness

Confining our analysis to respondents in just 11 days may have introduced bias, potentially overrepresenting frequent or regular users. We did not require users to complete the online survey before accessing the website, so respondents were self‐selected. We presented the survey as an opportunity to help improve Reflora resources, which may have made regular users more inclined to respond (possibly because they were more likely to consider their input useful) and perhaps more motivated to enhance a resource they found useful.

Maximizing inclusion was a primary consideration in our literature search, and it prompted us to use Google Scholar rather than other bibliographic resources that support more flexible, structured searches but in which Brazilian publications and gray literature are underrepresented. Similarly, we considered publications in which *reflora* appeared anywhere, rather than focusing exclusively on the subset specifying the particular use of Reflora resources. Nonetheless, we inevitably missed papers that merited inclusion. For example, researchers in the Reflora research and mobility program varied in their diligence in acknowledging Reflora when publishing, so publications in which acknowledgements include Reflora probably underrepresent total publications attributable to the Reflora program. This underrepresentation is dwarfed by another factor that became apparent during the preparation of this paper. Several of us who are regular RVH users and authors of scientific publications, including conservation assessments of Brazilian plants, regularly fail to cite RVH when we ought to. Consultations of RVH early in a study, for example, to locate material, tend to be forgotten by the time publications are being finalized and sources cited. All these lines of evidence suggest we probably underestimated the use of Reflora resources for conservation purposes.

### Reliability of Results

Our online survey had multiple choice questions followed by open questions that called for free‐text answers. This approach allowed respondents to describe in their own words how they used Reflora and avoided the need for us to anticipate what the most frequent uses might be. Disadvantages included the time required to read and interpret responses and potential subjectivity in mapping respondents’ descriptions of use to a common set of tags. Tagging literature‐search results was even more time consuming because there was more text. On balance, our approach was suitable for exploratory analysis of resource use but probably not appropriate for ongoing monitoring because costs would be prohibitive. However, categories of use resulting from our study could prove relevant to designing automated monitoring approaches in the future.

### Comparison with other Studies

To our knowledge, few other VHs have been created with a primary conservation focus, but useful comparators include large VHs reporting recent surveys that cover conservation use. Our approach to analyzing RVH use contrasts with that of the large and long‐established AVH (Cantrill 2018), which requires users requesting downloads to select from given options the purpose for which data are requested. Advantages of AVH's approach include less time required to interpret use, but a disadvantage is that about one‐third of users choose general categories such as “other,” which provides little information on why they are using the AVH. Cantrill (2018) considered AVH downloads for conservation management, environmental assessment, and restoration or remediation purposes as conservation activities and as distinct from downloads for scientific research (systematics, ecology, and other). Based on this information, conservation‐relevant research cannot be quantified. Similarly, survey results from Brazil's National Institute for Science and Technology Virtual Herbarium include conservation as 1 of 7 research fields that collectively account for 43% of use, but endangered species research is under another category (Maia et al. 2017) so total use for conservation cannot be determined.

### Learning from the Reflora Experience

Although Reflora is widely and justifiably recognized as a very successful program, our results highlight significant scope for improvement, which may be of interest to those planning a conservation‐focused VH.

Despite being conceived and funded with an explicit focus on supporting conservation, the RVH strongly resembles other VHs in aspect and functionality, and its special purpose in supporting conservation is not apparent to the general user of the Reflora resources. Researchers co‐located with CNCFlora at JBRJ know that the RVH is a key source of data for species conservation assessments and area prioritization for conservation and that conservation products relying on Reflora data are regularly commissioned by decision makers at state and federal levels (G. Martinelli, personal communication). However, the conservation relevance of the RVH is not as evident from the scientific literature as one might expect, perhaps because much of the conservation assessment, analysis, and dissemination takes place in other systems, so the value chain from specimen data to conservation product is difficult to track or discern. Thus, highlighting conservation as a primary objective of a new VH is not sufficient to maximize conservation relevance, impact, and profile.

A conservation focus should be mainstreamed throughout the VH, from development of the specification to the success criteria by which it will be judged. Preparation of detailed use scenarios by conservation scientists should be undertaken at the planning stage to complement uses that mirror those of physical herbaria. Where it proves impossible to deliver all desired conservation‐relevant functionality within the VH itself, highest priority should be given to maximizing integration or interoperability with systems in which VH data will be analyzed for conservation purposes. An emphasis on bidirectional data flows is essential because conservation analyses are often iterative processes in which preliminary analyses reveal errors or inconsistencies in data that are then refined before further analyses. The RVH was designed with the expectation that data would be improved over time and maintains clear separation between originally transcribed data and revised data fields for corrected or refined data. Where subsets of VH data are upgraded for analysis, VH curators, and conservation scientists must demonstrate a shared commitment to ensure that enhanced data are transmitted to the VH and incorporated, where appropriate. Mechanisms to facilitate such data flows should enable changes to VH data at different scales, from correction of a single field in a single record (included in the design of RVH from the outset) to data sets involving many thousands of records in which different fields have been changed (an increasingly frequent situation for which RVH lacks a mechanism). Moving beyond linking data sets to increased integration will be vital if scarce conservation resources are to be used for maximum impact.

For a conservation‐focused VH, demonstrating success needs to go beyond the metrics typical of other VHs: number of specimens incorporated, number of user consultations, and number of downloads. Important though these statistics are, they must be complemented by a more detailed and nuanced account of exactly how VH data are used for conservation purposes, including uses presented by geographic area, taxonomic group, and nature of the conservation impact. Mechanisms to gather at least some of these data need to be in place at VH launch and will probably need refining as VH use increases and evolves. Although capture and analysis of such metadata may be seen as an undesirable additional expense, their value should not be underestimated because the ability to quantify conservation impacts will be important to the sustainability of VHs. Numbers are necessary, but stakeholders beyond the immediate user community will find stories more compelling and memorable in campaigns to convince them of the centrality of VH in plant conservation and the importance of continued funding to guarantee ongoing availability. Those who consider a VH of central importance to plant conservation need to develop a portfolio of concise cases that exemplify clearly how actions to conserve certain species have been informed by data from a VH. If the species in question is attractive or unusual and the specimen data required expert interpretation, then so much the better, but the vital point is that the story starts with the specimens, without which tropical conservation biologists cannot know which species are of potential concern, where to look for them, and how to identify them.

## Supporting information

Details of literature review including tags (Appendix S1), online survey (Appendix S2), and conservation category spectra (Appendix S3) are available online. The authors are solely responsible for the content and functionality of these materials. Queries (other than absence of the material) should be directed to the corresponding author.
