# Supplementary material for: Enhancement of conservation knowledge through increased access to botanical information
Source: Conserv Biol. 2019 Feb 26;33(3):523–33. doi: 10.1111/cobi.13291 (PMC6850347; doi:10.1111/cobi.13291)
Supplement: Supplementary file 1 — Details of literature review including tags (Appendix S1), online survey (Appendix S2), and conservation category spectra (Appendix S3) are available online. The authors are solely responsible for the content and functionality of these materials. Queries (other than absence of the material) should be directed to the corresponding author. [file COBI-33-523-s001.docx]

**Supporting Information**

**Article: Enhancement of conservation knowledge through increased access to botanical information**

**Appendix S1**

**Literature Review Methodology**

On the 18^th^ September 2017, we conducted a Google Scholar search using the Publish or Perish 5 software (Harzing 2007) with the search term “reflora”. All publications prior to 2010 were excluded from analysis as this was prior to the establishment of the Reflora program. The returned publications were then split into different spreadsheet tables, by publication year, for individuals to analyze and score. All publications were downloaded prior to scoring.

*First stage paper data collection steps*

1. If the publication was in Portuguese the column headed Portuguese in the spreadsheet was marked with a Y so the next steps were completed by a Portuguese speaker. The remainder of the review team were competent to evaluate papers in English, French and Spanish. Papers also excluded from further analysis.
2. A search was run on the text of the whole publication to find which section of the document reflora was mentioned. A drop-down list was then used to populate a column headed Where mentioned in the spreadsheet. However, free text was used if nothing suitable was listed, or if the word reflora appeared in more than one section. The values used were Abs (abstract), Ack (acknowledgements), Appendix, Concl (conclusion), Discus (discussion), Figs (figures), Intro (introduction), Lit (references), Litrev (literature review), Main (Main text), Meth (methods) and Res (results). If the publication was not found to be related to the Reflora project it was marked for exclusion.
3. A search was run on the text of the whole publication for the search term “conserv”. If the search term was not found the Conserv column of the scoring spreadsheet was marked as NA. If the search term was found and the publication was relevant to conservation the publication was marked as Conserv. If the search term was found but the publication wasn’t relevant to conservation it was marked as ExclCons. If it was unclear if the publication was about conservation or not it was marked as Uncertain, which flagged the publication as a record for another individual to check. All papers marked as Conserv were selected for further analyze in stage two.
4. The main focus of the publication was determined from reading the title and the abstract, each publication was tagged with one or more focus terms. If the publication had already been tagged as a Conserv publication it was not compulsory to fill in this focus field as these publications were read and analyzed in greater depth in stage two for further categorization. Publications were specifically tagged if they were about new species, new genus, a rediscovery or a first record for a country or state. Any unexpected or novel publications were also tagged at this stage. Where possible the main focus term(s) was selected from a drop-down list. However, if nothing was suitable free text was allowed. All tags were discussed and consolidated further and categorized once all publications had been scored during this first phase.
5. The type of publication was determined. Publications other than scientific journal papers were marked accordingly e.g. PhD thesis, MSc thesis, posters and conference proceedings

To assess the consistency of scoring between individuals a Fleiss’ kappa test was performed. All reviewers evaluated a random set of 25 publications, following the process described above. The kappa statistic was calculated to measure the level of agreement between reviewers. A score of 0.65 was achieved on the first round however, after further discussion on standardizing focus terms to use and assessing conservation relevance, a score of 0.77 was achieved on the second round, which represents a substantial level of agreement (Landis & Koch 1977). Reviewers were then assigned subsets of the publications to analyze, consistent with their language skills and scientific experience.

*Second stage publication data collection steps*

1. The following information was collected for all New Species/New Genera: Family, Taxon name, Country and State(s) where found, Type locality, Date of first collection, if a conservation assessment has been completed and IUCN category where available. It was also noted if any of the specimens were found in a protected area.
2. The following information was collected for all First records: Family, Taxon name, Country and state(s) where specimens were found for the first time.
3. The following information was collected for all rediscoveries: Family, Taxon name, State re-found, Year rediscovery publication published, Year that species was described, Date of type collection, Conservation assessment and IUCN category, Conservation notes, Year last seen and Date rediscovered.
4. All publications where the term reflora was found in the methods section text were analyzed to determine which reflora resource was consulted – the Virtual Herbarium or the Flora of Brazil 2020 –, what information was gathered from it and how that information was used. For example, it was noted if Reflora was used for identification purposes, to find distribution or nomenclature information and if specimens were consulted.
5. All publications that were identified as conservation relevant publications in the first stage were read and analyzed further to determine the main focus of the publication. The publications were again categorized and tagged. Where possible a drop-down list was used but a free text was allowed when no suitable categories existed. After scoring of all publications tags were discussed and consolidated.
6. All publications that were marked as interesting or novel were read in full. A short summary of contents was noted along with what was particular of interest and how reflora was used in the publication.


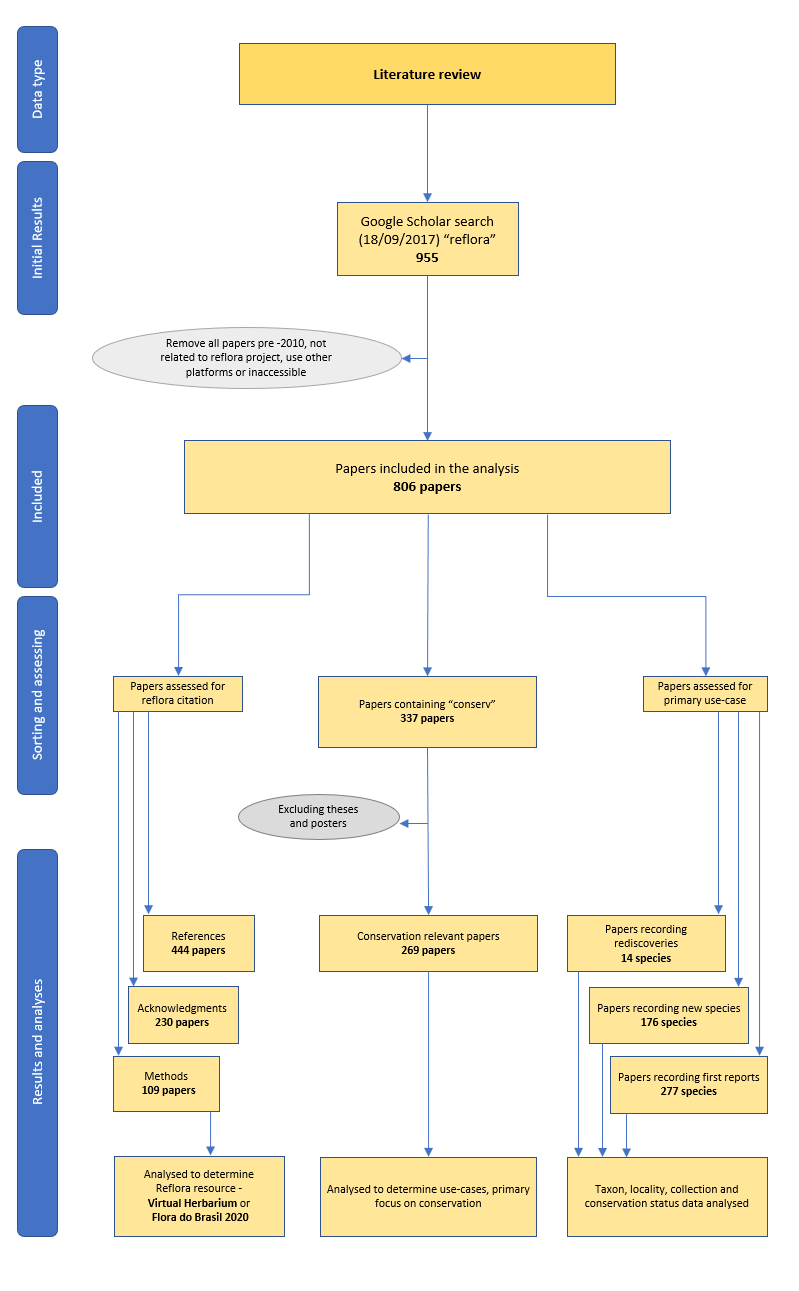


Figure S1.1: Summary of analyses undertaken in the review of the literature to ascertain conservation-relevance and use-cases of papers citing Reflora.

**Research Topics Tags**

Below is the list of main research topics reported in the online survey and in the published scientific literature and a more detailed description of the types of research, studies and uses that come under these tags.

**Distribution**

This covers all online survey responses and publications pertaining to biogeography, distribution frequency.

**Chemistry**

This covers all online survey responses and publications pertaining to chemistry.

**Climate Change**

This covers all online survey responses and publications pertaining to climate change.

**Conservation Assessment**

This covers all online survey responses and publications where a conservation risk assessment of species or habitat(s) was undertaken.

**Conservation Genetics**

This covers all online survey responses and publications pertaining to conservation genetics and phylodiversity conservation.

**Conservation Planning & Prioritisation**

This covers all online survey responses and publications pertaining to conservation planning, conservation priorities, conservation implications and unprotected areas.

**Conservation Status**

This covers all online survey responses and publications citing conservation status of species or ecosystems based on work already reported elsewhere e.g. publications in which for each species mentioned a previously published IUCN Red List assessment is cited, if available or online respondents reporting use of Reflora resources to check the conservation status of a species already evaluated.

**Digitisation & Data Infrastructure**

This covers all online survey responses and publications pertaining to digitization, data infrastructure, Information Technology, Herbarium reports.

**Ecology**

This covers all online survey responses and publications pertaining to areas in ecology such as autecology, paleoecology, pollination, frugivory, agrosystems.

**Education & Dissemination**

This covers all online survey responses and publications pertaining to education, information dissemination, websites, ecotourism and contact with experts.

**Endemism**

This covers all online survey responses and papers pertaining to endemic species and levels of endemism.

**Environmental Assessment & Monitoring**

This covers all online survey responses and publications pertaining to environmental impact assessments, environmental monitoring, habitat quality evaluation, bioindicators.

***Ex situ* conservation**

This covers all online survey responses and publications pertaining to *ex situ* conservation, including seed banking and seed collecting.

**Field Work**

This covers all online survey responses and publications pertaining to field work, field work planning, field collecting and expeditions.

**First Records**

This covers all online survey responses and publications pertaining to first records of taxa for a particular area e.g. country, Brazilian state or region or phytogeographic domain

**Floras & Field Guides**

This covers all online survey responses and publications pertaining to Floras, field guides, checklists, keys for use in the field.

**Floristics**

This covers all online survey responses and publications pertaining to floristics, vegetation, vegetation surveys, phytosociology, vegetation classification, and studies of a similar nature but with a focus on fungi rather than plants

**Forest Inventory**

This covers all online survey responses and publications pertaining to forest inventories, mainly for the project Inventário Florestal Nacional.

**Horticulture**

This covers all online survey responses and publications pertaining to horticulture.

**Identification**

This covers all online survey responses and publications that used the resources to help with species identification.

**Invasives**

This covers all online survey responses and publications pertaining to invasive species.

**Management Planning**

This covers all online survey responses and publications pertaining to management planning, recommendations and considerations.

**Morphology**

This covers all online survey responses and publications pertaining to morphology, micromorphology, anatomy, palynology.

**New Species**

This covers all online survey responses and publications pertaining to new species, new genera, new hybrids.

**Phenology**

This covers all online survey responses and publications pertaining to phenology and conservation biology.

**Plant Propagation**

This covers all online survey responses and publications pertaining to plant propagation (non-economic).

**Policy**

This covers all online survey responses and publications pertaining to conservation policy, policy making, law and protocols such as the Nagoya protocol.

**Population**

This covers all online survey responses and publications pertaining to populations, population dynamics, population census.

**Protected Areas**

This covers all online survey responses and publications pertaining to protected areas.

**Rare/ Threatened Species**

This covers all online survey responses and publications pertaining to threatened species, rare species and rarity types.

**Rediscoveries**

This covers all online survey responses and publications pertaining to rediscoveries of species which had not been seen for a protracted period of time

**Reintroductions**

This covers all online survey responses and publications pertaining to reintroductions.

**Reproductive Biology**

This covers all online survey responses and publications pertaining to reproductive biology.

**Restoration, Remediation & Reforestation**

This covers all online survey responses and publications pertaining to restoration, remediation, reforestation, water treatment.

**Seed Biology**

This covers all online survey responses and publications pertaining to seed biology, seed physiology and spore germination.

**Spatial Analysis**

This covers all online survey responses and publications pertaining to spatial analysis, species distribution modelling, sample gap analysis.

**Species Origin**

This covers all online survey responses and publications pertaining to species origin, native species and exotic species.

**Taxonomy and Phylogeny**

This covers all online survey responses and publications pertaining to taxonomy, phylogeny, nomenclature, synonymy, new combinations and re-establishment species previously treated in synonymy but now recognized as distinct.

**Useful Plants**

This covers all online survey responses and publications pertaining to useful plants, plant health, economic use, ethnobotany, medicinal use, wild harvested species, forage, beekeeping.

**Zoology**

This covers all online survey responses and publications pertaining to zoology.

**Other**

This covers all online survey responses and publications pertaining to other research areas such as collection management strategies, history, translations, fund raising and other categories that would not fit elsewhere.

**Appendix S2**

A link to the online survey below was shared for one month on the Reflora homepage (reflora.jbrj.gov.br/, Fig. S1) and the Virtual Herbarium (<http://reflora.jbrj.gov.br/reflora/herbarioVirtual/>, Fig. S2) at points visible to both casual and logged in users, as well as on the log-in page of the system used mainly by contributors to the Flora of Brazil 2020 in both Portuguese and English. We wanted to reach all users of the Virtual Herbarium, but not necessarily all those accessing the Flora. However, the way both systems are integrated did not allow a complete differentiation between them.


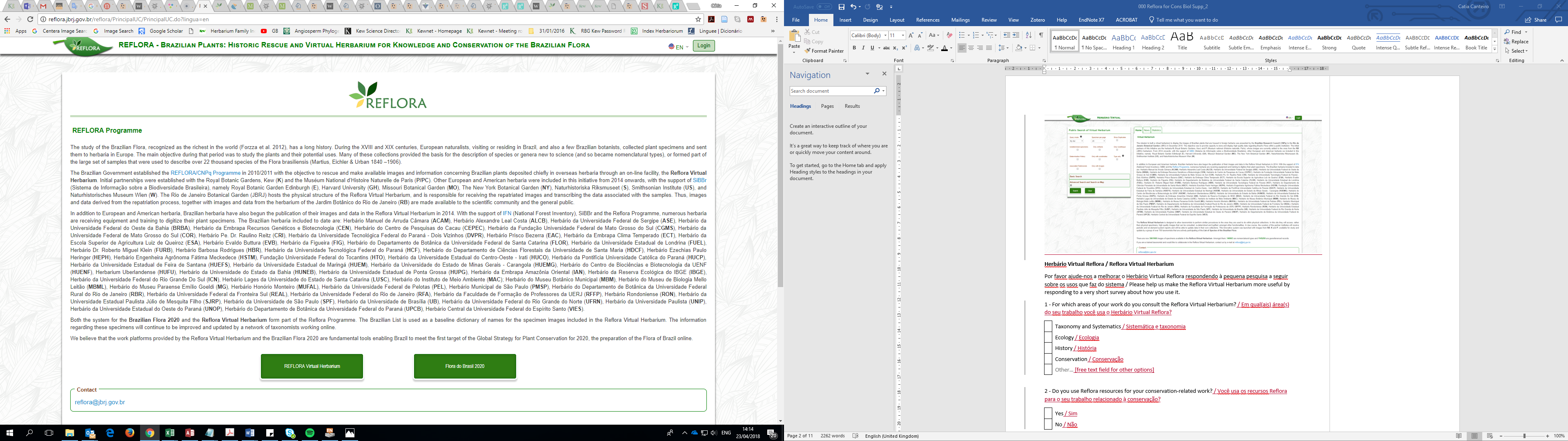


Figure S1.1: Screenshot of the Reflora homepage (<http://reflora.jbrj.gov.br/>), one of the pages where the link to the survey was shared.


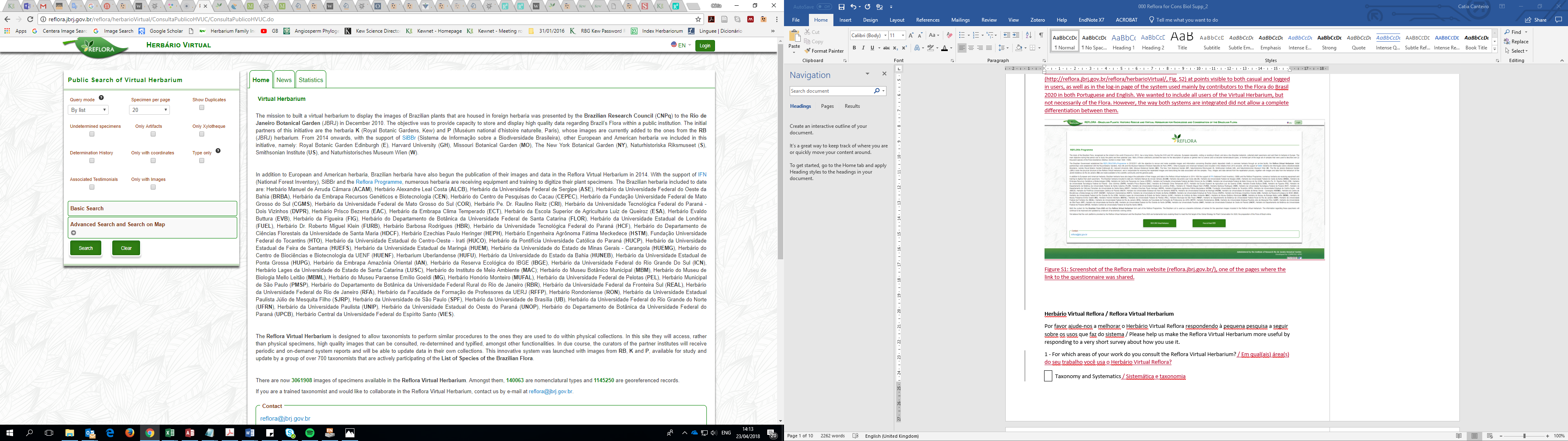


Figure S1.2: Screenshot of the Reflora Virtual Herbarium webpage (<http://reflora.jbrj.gov.br/reflora/herbarioVirtual/>), one of the pages where the link to the survey was shared, as regular users of the RVH often enter via a saved link to this page rather than via the Reflora homepage above.

**Wording of the online survey in Portuguese and in English**

**Herbário Virtual Reflora / Reflora Virtual Herbarium**

Por favor ajude-nos a melhorar o Herbário Virtual Reflora respondendo à pequena pesquisa a seguir sobre os usos que faz do sistema / Please help us make the Reflora Virtual Herbarium more useful by responding to a very short survey about how you use it.

1 - For which areas of your work do you consult the Reflora Virtual Herbarium? / Em qual(ais) área(s) do seu trabalho você usa o Herbário Virtual Reflora?

|  | Taxonomy and Systematics / Sistemática e taxonomia |
| --- | --- |
|  | Ecology / Ecologia |
|  | History / História |
|  | Conservation / Conservação |
|  | Other… [free text field for other options] |

2 - Do you use Reflora resources for your conservation-related work? / Você usa os recursos Reflora para o seu trabalho relacionado à conservação?

|  | Yes / Sim |
| --- | --- |
|  | No / Não |

3a - Your use of Reflora resources for your conservation-related work was an intentional search (you already knew Reflora could be used for this purpose) or was it occasional? / O uso dos recursos Reflora para o seu trabalho relacionado à conservação foi uma busca intencional (já sabia que o Reflora poderia lhe propiciar este uso) ou foi ocasional?

|  | Intentional / Intencional |
| --- | --- |
|  | Occasional / Ocasional |

4 - Please give us an example of your use of Reflora resources for your conservation-related work. A short description and/or a reference to a publication would be useful. / Por favor nos dê um exemplo de como você usa os recursos do Reflora para o seu trabalho em Conservação. Uma pequena descrição e/ou uma referência para uma publicação serão úteis.

3b - Please say why you don’t use Reflora resources for your conservation-related work. / Por favor diga os motivos porque você não usa os recursos do Reflora para o seu trabalho em Conservação.

|  | 1 - For which areas of your work do you consult the Reflora Virtual Herbarium? | | | | 2 - Do you use Reflora resources for your conservation-related work? | | 3a - Your use of Reflora resources for your conservation-related work was an intentional search (you already knew Reflora could be used for this purpose) or was it occasional? | |
| --- | --- | --- | --- | --- | --- | --- | --- | --- |
|  | Taxonomy | Ecology | Conservation | History | Yes | No | Intentional | Occasional |
| Responses up to 20/10/2017 | 43% | 23% | 22% | 4% | 81% | 19% | 83% | 17% |
| Responses between 20/10/2017 and 08/11/2017 | 37% | 26% | 22% | 4% | 76% | 24% | 74% | 26% |
| All responses | 47% | 27% | 16% | 3% | 79% | 21% | 80% | 20% |

Table S1.1: Comparison of responses for the questions number 1, 2 and 3a of the online survey for the 11-day period analyzed in our paper and the remaining period not analyzed.

| Category | Total | Proportion of all tags | Proportion of answers |
| --- | --- | --- | --- |
| Taxonomy & Phylogeny | 831 | 42.6% | 77.7% |
| Ecology | 446 | 22.9% | 41.7% |
| Conservation | 435 | 22.3% | 40.7% |
| History | 72 | 3.7% | 6.7% |
| Other | 42 | 2.2% | 3.9% |
| Education | 16 | 0.8% | 1.5% |
| Morphology | 16 | 0.8% | 1.5% |
| Distribution | 15 | 0.8% | 1.4% |
| Useful Plants | 14 | 0.7% | 1.3% |
| Environmental Assessment & Monitoring | 11 | 0.6% | 1.0% |
| Identification | 11 | 0.6% | 1.0% |
| Restoration, Remediation & Reforestation | 11 | 0.6% | 1.0% |
| Floristics | 8 | 0.4% | 0.7% |
| Plant Propagation | 6 | 0.3% | 0.6% |
| Chemistry | 4 | 0.2% | 0.4% |
| Digitisation & Data Infrastructure | 4 | 0.2% | 0.4% |
| Horticulture | 4 | 0.2% | 0.4% |
| Conservation Planning & Prioritisation | 1 | 0.1% | 0.1% |
| Management Planning | 1 | 0.1% | 0.1% |
| Species Origin | 1 | 0.1% | 0.1% |
| TOTAL | 1949 |  |  |

Table S1.2: Areas of work included in the responses for question 1 of the online survey.

| Category | Total | Proportion of all tags | Proportion of answers |
| --- | --- | --- | --- |
| Distribution | 368 | 25.9% | 43.9% |
| Taxonomy & Phylogeny | 187 | 13.2% | 22.3% |
| Identification | 165 | 11.6% | 19.7% |
| Conservation Status | 114 | 8.0% | 13.6% |
| Floristics | 59 | 4.2% | 7.0% |
| Conservation Assessment | 51 | 3.6% | 6.1% |
| Protected Areas | 49 | 3.5% | 5.8% |
| Rare/ Threatened Species | 49 | 3.5% | 5.8% |
| Endemism | 47 | 3.3% | 5.6% |
| Morphology | 39 | 2.7% | 4.7% |
| Species Origin | 37 | 2.6% | 4.4% |
| Restoration, Remediation & Reforestation | 33 | 2.3% | 3.9% |
| Plant Propagation | 29 | 2.0% | 3.5% |
| Useful Plants | 23 | 1.6% | 2.7% |
| Education & Dissemination | 22 | 1.5% | 2.6% |
| Environmental Assessment & Monitoring | 18 | 1.3% | 2.1% |
| Management Planning | 16 | 1.1% | 1.9% |
| Other | 16 | 1.1% | 1.9% |
| Spatial Analysis | 14 | 1.0% | 1.7% |
| Floras & Field Guides | 13 | 0.9% | 1.6% |
| New Species | 12 | 0.8% | 1.4% |
| Forest Inventory | 9 | 0.6% | 1.1% |
| Horticulture | 7 | 0.5% | 0.8% |
| Field Work | 6 | 0.4% | 0.7% |
| Phenology | 6 | 0.4% | 0.7% |
| Zoology | 6 | 0.4% | 0.7% |
| Ex Situ Conservation | 5 | 0.4% | 0.6% |
| Population | 5 | 0.4% | 0.6% |
| Ecology | 4 | 0.3% | 0.5% |
| Climate Change | 3 | 0.2% | 0.4% |
| Invasives | 2 | 0.1% | 0.2% |
| Reintroductions | 2 | 0.1% | 0.2% |
| Conservation Genetics | 1 | 0.1% | 0.1% |
| Conservation Planning & Prioritisation | 1 | 0.1% | 0.1% |
| Policy | 1 | 0.1% | 0.1% |
| Rediscoveries | 1 | 0.1% | 0.1% |
| Total | 1420 |  |  |

Table S1.3: Areas of work included in the responses for question 4 of the online survey.

**Appendix S3**

**
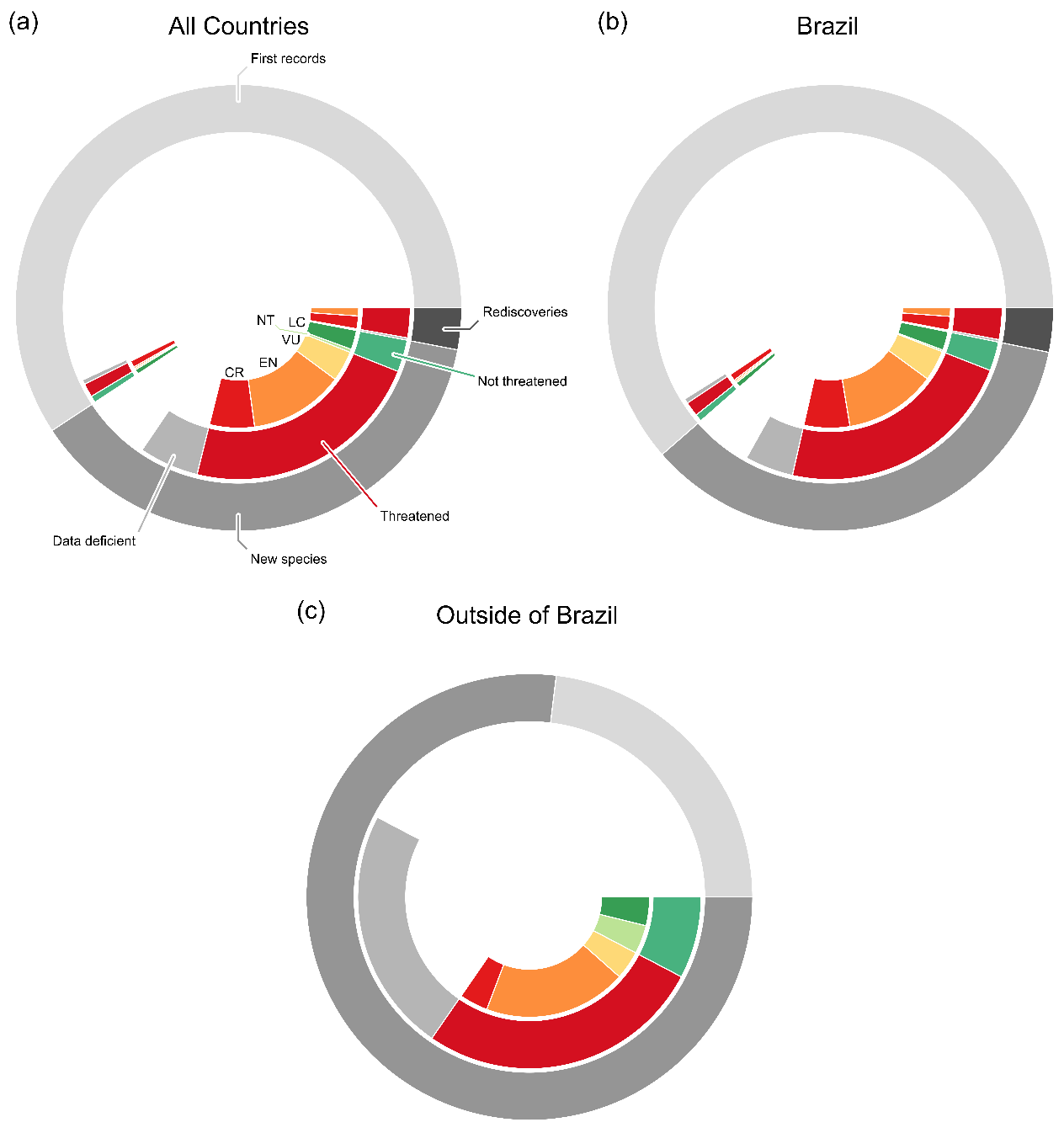
**

Figure S3.1: (a) Breakdown of all taxa identified in the literature review as new species, rediscovered species, and first reports (outer ring), with the proportions of each that were assessed as threatened, not threatened, and data deficient (middle ring). These are further broken down, (inner circle), into species categorized as least concern (LC), near threatened (NT), vulnerable (VU), endangered (EN), and critically endangered (CR). Subsets of the data comprising (b) any species found in Brazil and (c) species only found outside of Brazil show similar patterns.

**References**

Harzing A-W. 2007. Publish or Perish, Available from http://www.harzing.com/pop.htm (accessed September 2017.

Landis JR, Koch GG. 1977. The measurement of observer agreement for categorical data. Biometrics 33:159-174.
